# Estimating the Pollution Risk of Cadmium in Soil Using a Composite Soil Environmental Quality Standard

**DOI:** 10.1155/2014/750879

**Published:** 2014-02-04

**Authors:** Mingkai Qu, Weidong Li, Chuanrong Zhang, Biao Huang, Yongcun Zhao

**Affiliations:** ^1^Key Laboratory of Soil Environment and Pollution Remediation, Institute of Soil Science, Chinese Academy of Sciences, Nanjing, Jiangsu 210008, China; ^2^Department of Geography and Center for Environmental Sciences and Engineering, University of Connecticut, Storrs, CT 06269, USA

## Abstract

Estimating standard-exceeding probabilities of toxic metals in soil is crucial for environmental evaluation. Because soil pH and land use types have strong effects on the bioavailability of trace metals in soil, they were taken into account by some environmental protection agencies in making composite soil environmental quality standards (SEQSs) that contain multiple metal thresholds under different pH and land use conditions. This study proposed a method for estimating the standard-exceeding probability map of soil cadmium using a composite SEQS. The spatial variability and uncertainty of soil pH and site-specific land use type were incorporated through simulated realizations by sequential Gaussian simulation. A case study was conducted using a sample data set from a 150 km^2^ area in Wuhan City and the composite SEQS for cadmium, recently set by the State Environmental Protection Administration of China. The method may be useful for evaluating the pollution risks of trace metals in soil with composite SEQSs.

## 1. Introduction

With increasing industrialization and rapid urbanization in many regions of the world, contamination by trace metals in the terrestrial environment has become widespread in a global context [1]. Accumulation of trace metals such as cadmium (Cd) in soil may impact soil quality, reduce soil biological activity, and hinder the effective supply of nutrients. More importantly, trace metals can be largely enriched through the food chain and other ways, directly or indirectly threatening human health [2]. Therefore, environmental protection agencies often set soil environmental quality standards (SEQSs) for trace metal concentrations in soil, and the standards may have the force of law to land management and environmental remediation.

An important aim of many soil environmental surveys is to delimit the zones potentially contaminated by toxic metals, for which we need to know the spatial distribution of a toxic metal in soil in the surveyed area. Over the last 20 years, spatial interpolation techniques, such as kriging, have been often used to interpolate the spatial distributions of trace metals in soil and further delineate their standard-exceeding areas [3, 4]. However, the smoothing effect, commonly found in the maps generated by interpolation methods, often results in less variation in the estimated values than in the observed values [5, 6]. It is widely known that this problem causes low values to be overestimated and high values to be underestimated. There is always some uncertainty associated with an optimal estimate by kriging or indeed any other form of interpolation, and a decision made purely on the basis of such an estimate carries with it the risk that an unsampled site is declared “safe” where the soil is toxic or “toxic” where it is not [7]. There is an increasing awareness that an estimate is more valuable in the presence of a measure of the associated uncertainty, and this is particularly the case in prediction of environmental variables where the prediction uncertainty is required to support decision-making about further management [8]. Sequential simulation methods, such as sequential Gaussian simulation (SGS), provide a useful solution for this problem, because simulated realizations overcome the smoothing effect and spatial uncertainty measures such as threshold-exceeding probabilities can be estimated from a number of simulated realizations [5, 9]. In addition, interpolated values at unsampled locations never exceed the limits of maximum and minimum values of samples within corresponding neighborhoods, but simulated values by SGS are not limited by the bounds.

In environmental modeling, estimating the probability of a trace metal exceeding a threshold (or cutoff) value is an effective method for spatial (i.e., site-specific) uncertainty assessment, which is crucial for environmental evaluation and decision making [5, 10–12]. Zones may then be delineated and ranked according to the probabilities with which the unknown concentrations of a pollutant exceed the threshold at all locations in a study area. If remediation or further sampling is needed then they should be done first in the zones with the largest probabilities of contamination. There are a variety of spatial statistical approaches which may be used for estimating threshold-exceeding probabilities, such as indicator kriging [13], disjunctive kriging [14], generalized linear model [15], clipped Gaussian random fields [16], sequential indicator simulation [6], sequential Gaussian simulation [17], and Markov chain random fields [18]. However to the best of our knowledge, earlier studies on the estimation of threshold-exceeding probabilities were mainly based on a single threshold. It is known that soil pH has strong impact on the bioavailability (or toxicity) of some trace metals in soil due to its effects on the solubility from mineral surfaces, movement, and speciation of the trace metals both in soil bodies and particularly in soil solutions [19, 20]. Moreover, toxic metals may have different hazards to receptors (e.g., plants) in different land use types. Therefore, soil pH and land use type are two important factors that should be considered by policy makers besides the total amounts of trace metals. In fact, these two factors have been taken into account by some government agencies in making SEQSs of trace metals in soil. For example, the State Environmental Protection Administration of China [21] and the Consejeria de Medio Ambiente de la Junta de Andalucía (environmental agency of the regional government of the South of Spain) [22] considered soil pH and land use type information in making their SEQSs for some trace metals. Thus, the SEQS for a specific trace metal becomes a composite one; that is, it is composed of multiple threshold values under different conditions, rather than a single threshold value. In this study, the SEQS that we used for soil Cd is composed of a series of concentration thresholds with corresponding pH value intervals and land use types. It is apparent that spatial uncertainty is an intrinsic characteristic in the spatial distributions of soil trace metals and pH due to limited observation points; thus, information of both uncertainties should be valuable to standard-exceeding probability estimation.

Sequential Gaussian simulation (SGS) is a widely used sequential simulation algorithm for continuous spatial variables. Its major purpose is to generate a number of simulated realizations of a target spatial variable in a study area, which can effectively reflect the spatial uncertainty of the target variable resulting from its spatial heterogeneity [23]. SGS has been frequently adopted to simulate the spatial patterns of contaminants in ground water and soils and delineate their probabilistic risks to surrounding environments [5, 23, 24]. In this study, SGS was used to simulate the spatial distributions of soil Cd and pH for evaluating their spatial variability and associated uncertainty in the study area, and both the sets of simulated realizations and site-specific land use type information were further used to estimate the standard-exceeding probability map of Cd in soil.

The objective of this study is to suggest a method for estimating the standard-exceeding probability map of soil Cd using a composite SEQS, that is, a method which incorporates both the spatial variability and uncertainties of soil Cd and pH and the site-specific land use type information. The method was demonstrated using a case study from an area in China. However, it is important to emphasize that the general approach can be applied to evaluate standard-exceeding probabilities of other environmental variables with a composite SEQS. To the best of our knowledge, so far this kind of study has not been seen in the literature.

## 2. Materials and Methods

### 2.1. Study Area and Data

A study area of approximately 150 km^2^ is chosen in the urban-rural transitional zone of the Wuhan City, a metropolis in the middle reach of the Yangtze River. The study area was mostly farmlands with villages previously, but it was arranged as a high-tech development park in late 1980s. Since then, some industrial companies were gradually established within the area.

**Figure 1:**
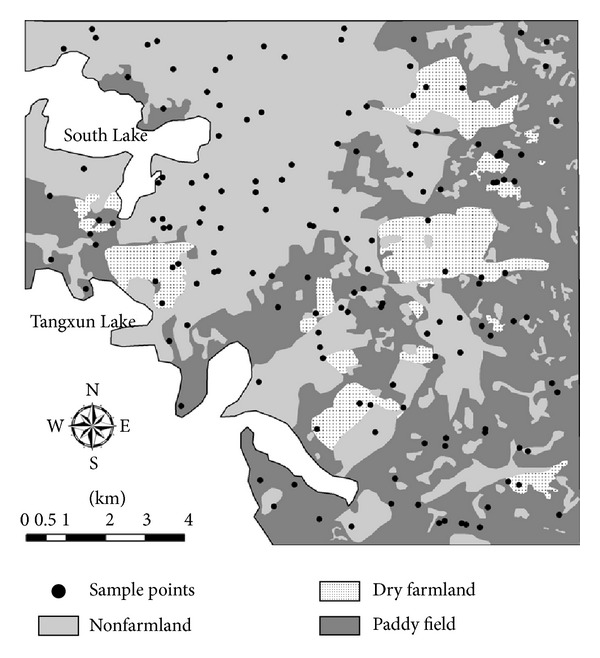
Soil sample locations and land use type distribution.

In this study, lands in the study area were divided into three types: *paddy field* referring to the arable land with water source and irrigation facilities and mainly used to grow rice, lotus root, and other aquatic crops; *dry farmland* referring to the arable land without water source and irrigation facilities and mainly used to grow wheat, corn, sesame, and other xeric crops; and *nonfarmland* referring to the nonarable land, mainly used for industry, business, transportation, and residence (see Figure 1).

An investigation of soils was performed in October, 2009. 150 nonrhizosphere topsoil samples (0–20 cm depth) were collected in the study area (see Figure 1), and the total concentration of Cd in soil and the pH value were measured for each soil sample. The coordinates of the sampling locations were georeferenced using a handheld GPS receiver (MAP60CSX, Garmin Ltd.). The procedures for soil sample preparation and lab analysis are as follows.

At each sampling point, 4–6 subsamples were randomly taken and then mixed to obtain a composite soil sample. All samples were air-dried at room temperature (20–22°C), crushed after stones and other debris were removed, and then sieved to 2 mm. A portion of each soil sample (about 50 g) was then ground in an agate grinder to the particle size of <0.149 mm (i.e., passing through a sieve of 1/100 mm meshes). The prepared soil samples were then stored in polyethylene bottles for later analysis. Soil samples were analyzed for measuring the total concentration of Cd and pH value. 0.5 g of each prepared soil sample was digested in a mixture of nitric acid (HNO_3_) and perchloric acid (HClO_4_) [25]; then the total concentration of Cd in the digested solution was measured using the inductively coupled plasma mass spectrometry (X7 ICP-MS, TMO, USA). The pH values of soil samples were measured in suspensions (soil: water = 1 : 2.5) with pH glass electrodes [26]. Quality assurance and quality control (QA/QC) for Cd in soil samples were estimated by determining the metal contents in blank and duplicate samples and standard reference materials.

### 2.2. Sequential Gaussian Simulation

The most comprehensive approach that can incorporate the variability of a soil attribute in space and also includes a measure of uncertainty is sequential simulation. This is a kind of geostatistical Monte Carlo methods whereby, instead of producing one map of best local estimates, the emphasis is on producing several feasible maps, of which each reasonably matches the sample statistics, variogram model, and conditioning (sample) data [5, 27]. Sequential Gaussian simulation is the most frequently used sequential simulation algorithm for simulating continuous variables. It assumes a Gaussian random field; thus, the Gaussian conditional cumulative distribution function (ccdf) of the studied variable is completely characterized by the mean value and covariance [28]. In SGS, simulation is conducted upon the Gaussian transformation of sample data if they obviously deviate from the Gaussian distribution. Detailed introduction on this method can be found in Goovaerts [5].

### 2.3. Standard-Exceeding Probability Assessment

In this study, the spatial distributions of soil Cd concentrations and pH values were simulated separately using SGS, and for each of them five hundred simulated realizations were generated. Thus, the uncertainty associated with each of Cd and pH estimates could be quantified using their respective simulated realizations. Then the standard-exceeding probability of soil Cd with the consideration of soil pH and land use type may be calculated. At a specific location **x**, the standard-exceeding probability of soil Cd can be estimated using the following equation with joint probabilities:

(1) P{[Z(x)>zc(x)] ∣ n}=∑i=1SP[αi(x)<zpHSGS(x)≤αi+1(x),  zCdSGS(x)>βi(x)],

where **Z**(**x**) is the concentration of soil Cd at location **x**; **z**_**c**_(**x**) refers to the composite SEQS for Cd with the conditions of pH and land use type at location **x**; *n* refers to the sample information for pH and soil Cd; *z*_pH_^SGS^(**x**) and *z*_Cd_^SGS^(**x**) are the simulated pH and Cd by SGS, respectively; *S* is the number of the segments of pH value range corresponding to different Cd thresholds for the SEQS (in this study *S* = 4); *α*_*i*_(**x**) and *α*_*i*+1_(**x**) are the low value and high value, respectively, for the *i*th value interval of pH in the SEQS; *β*_*i*_(**x**) is the Cd threshold corresponding to the pH interval of [*α*_*i*_(**x**), *α*_*i*+1_(**x**)].

**Figure 2:**
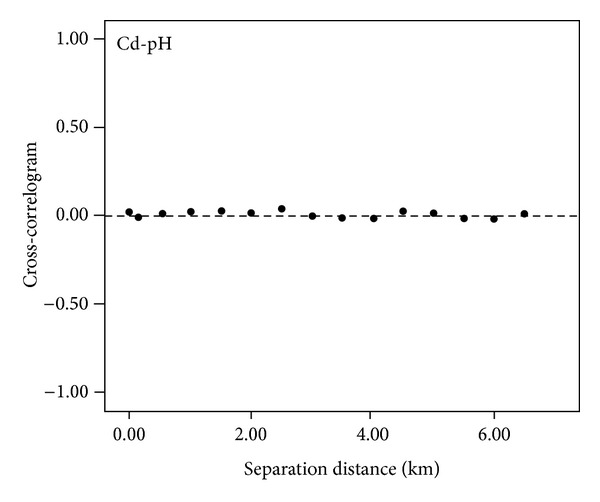
Cross-correlogram between Cd concentration and pH in soil.

Because the land use type is already known for a specific location (i.e., the land use map is available), it does not appear in (1). While Cd availability in soil is related to the value of soil pH, which entails the inclusion of soil pH in the SEQS of Cd, the total concentration of Cd in soil is mainly determined by other environmental factors (e.g., soil parental materials and pollution sources) rather than by soil pH. In addition, the cross correlogram between total Cd concentration and pH in soil (Figure 2) indicates that the assumption of independence between them is reliable. Thus, joint probability equation (1) can be simplified as

(2) P[Z(x)>zc(x) ∣ n]=∑i=1S{P[αi(x)<zpHSGS(x)≤αi+1(x)]   × P[zCdSGS(x)>βi(x)]},

with

(3) P[αi(x)<zpHSGS(x)≤αi+1(x)]  =N[αi(x)<zpHSGS(x)≤αi+1(x)]L,P[zCdSGS(x)>βi(x)]=N[zCdSGS(x)>βi(x)]L,

where *L* is the number of realizations generated by SGS for pH or Cd (in this study *L* = 500), and *N* is the number of realizations whose values at location **x** fall into the corresponding pH interval or Cd threshold.

The composite SEQS for soil Cd used in this study is listed in Table 1. The data of this composite standard for Cd in soil comes from the Soil Environmental Quality Standard II issued by the State Environmental Protection Administration of China [21]. The composite standard for soil Cd is considered for pH value intervals, and for each pH interval there are Cd threshold values corresponding to different land use types. For this study, we considered three land use types: paddy field, dry farmland, and nonfarmland. If the total concentration of Cd in soil exceeds the composite SEQS for soil Cd (i.e., exceeds the Cd threshold corresponding to the local soil pH and land use type) at a location, that means that the soil has been regarded as polluted at the location.

In this study, geostatistical simulation and standard-exceeding probability assessment were performed on a regular square grid of 60 m × 60 m.

## 3. Results and Discussions

### 3.1. Sample Data Analysis

A descriptive statistical summary of sample data for the concentrations of soil Cd and pH is listed in Table 2. The coefficient of variation (CV) implies low variability when it has a value of less than 10% and extensive variability when it is more than 90% [29]. The CVs for soil Cd and pH are 43.22% and 44.63%, respectively. This indicates that soil Cd and pH have moderate variability in the study area. The variations of Cd and pH may result from some extrinsic factors (such as industrial emissions) that are influential on soil Cd and pH. Soil samples tend to be acidic, with a mean pH value of 6.71. The mean concentration of Cd in soil samples exceeds the SEQS for paddy field and dry farmland when soil pH is less than 7.5 (see Table 1).

### 3.2. Spatial Distributions of Soil Cadmium and pH

**Figure 3:**
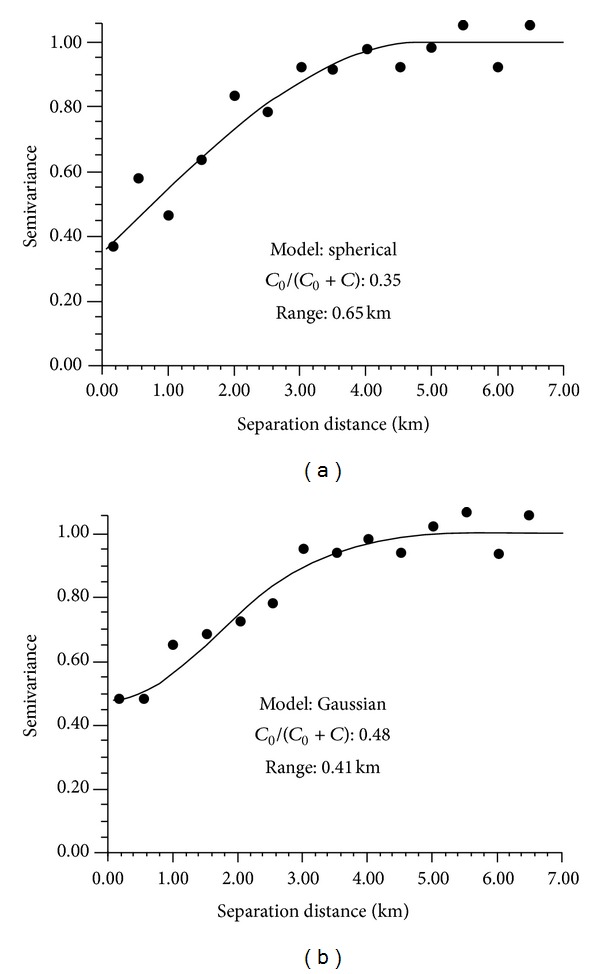
Experimental variograms of the normal score transformed data for soil Cd (a) and pH (b), with fitted models and parameters.

Experimental variograms of Cd and pH were estimated omnidirectly because of the lack of apparent anisotropy in sample data. Experimental variograms with fitted models and parameters for the normal score transformed data of soil Cd and pH are presented in Figure 3. The experimental variograms for normal score transformed Cd and pH data were fitted by a spherical model and a Gaussian model, respectively. The *C*_0_/(*C*_0_ + *C*) ratios of both fitted variogram models are between 25% and 75%, which exhibits moderate spatial autocorrelations. This situation may be attributed to both intrinsic factors such as soil properties and extrinsic factors such as human activities.

**Figure 4:**
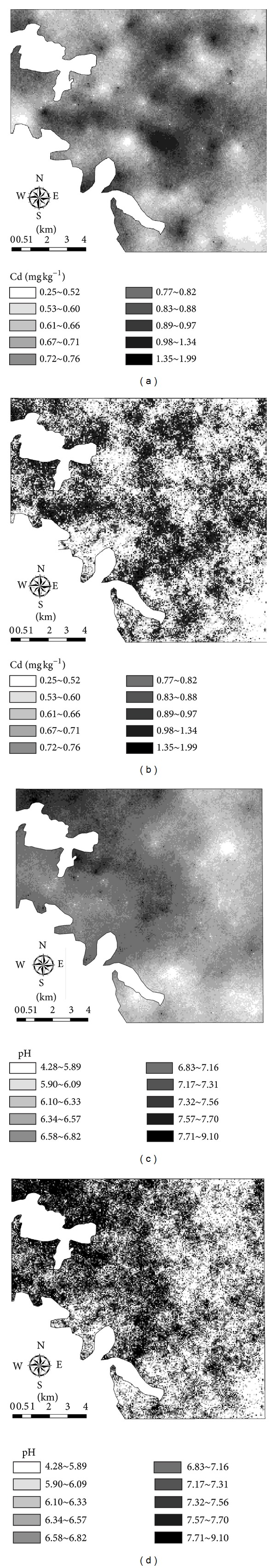
The E-type estimates and the 150th realizations generated by SGS for soil Cd (a) and (b) and pH (c) and (d), respectively.

The E-type estimate and a randomly selected realization generated by SGS for each of soil Cd and pH are shown in Figure 4. The E-type estimate map for each soil attribute was averaged from five hundred simulated realizations generated by SGS. While every simulated realization may represent a realistic spatial distribution of the corresponding soil attribute without the smoothing effect, the corresponding E-type estimate map does have the smooth effect and represents an optimal estimation. The Cd maps show that Cd has relatively high values in some small subareas in the central and north parts of the whole study area, which coincide with the locations of industry companies in the High-Tech Development Park of the city. The pH maps show that high values are concentratedly distributed in the north-west region, overlapping with the nonfarmland area. The fact that low pH values are mainly located in paddy fields and dry farmlands means that pH is strongly affected by agricultural practices.

### 3.3. Standard-Exceeding Probability Mapping

**Figure 5:**
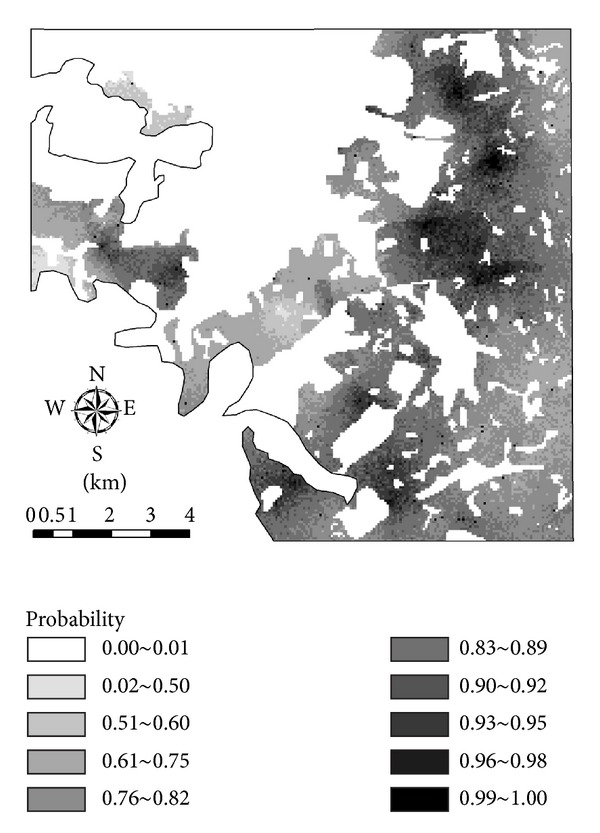
The probability map of Cd exceeding the composite soil environmental quality standard II of China for Cd in soil (referring to Table 1).

The probability map for soil Cd exceeding the composite environmental quality standard (Table 1) is presented in Figure 5. It can be seen that soil Cd basically does not exceed the standard in nonfarmland areas (see Figure 1 for land use types), where the exceeding probabilities are all less than 0.01. The major reason should be that the SEQS values of soil Cd set for the nonfarmland land use type are high (see Table 1). All farmlands (here farmlands include paddy fields and dry farmlands) have moderate to high pollution risks of Cd, but high risks (i.e., high standard-exceeding probabilities) mainly occur in those farmlands, particularly dry farmlands, along the interleaving zones of farmlands and nonfarmlands, where soil pH values are relatively low and industry companies are also distributed.

### 3.4. Cd Risk Area Delineation Based on Standard-Exceeding Probabilities

**Figure 6:**
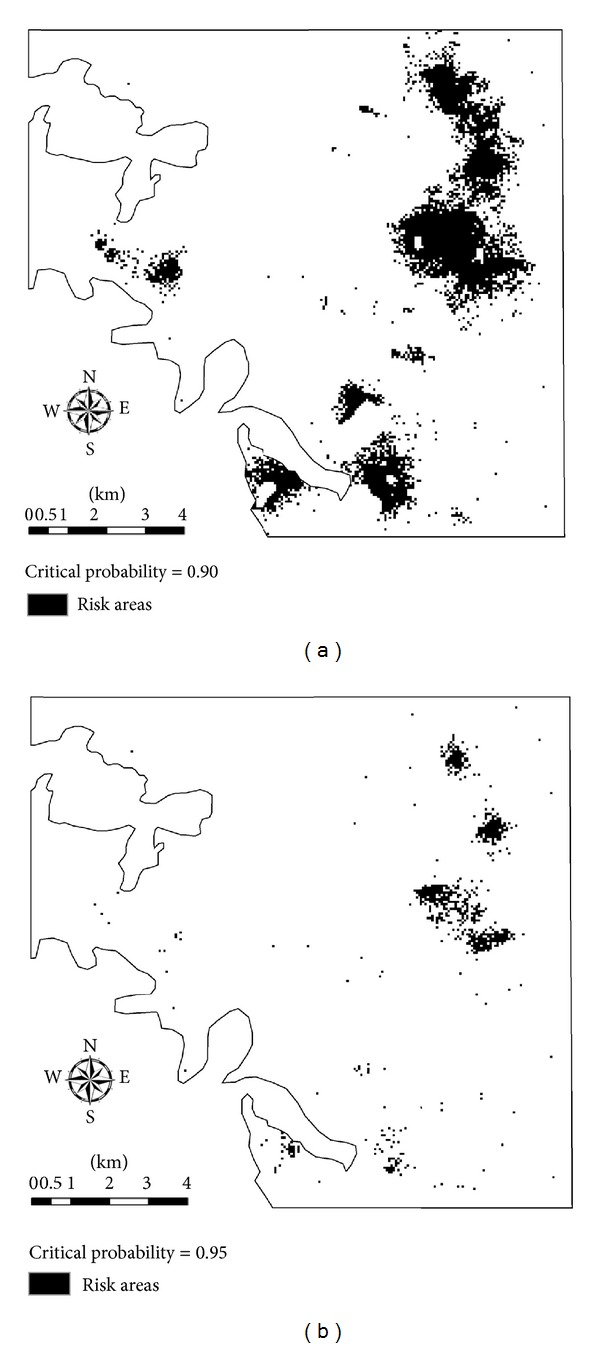
Risk areas based on the probability map of Cd exceeding the composite soil environmental quality standard II of China for Cd in soil given the critical probability values 0.90 (a) and 0.95 (b).

Remediation measures to Cd in soil should be first applied to those places with higher occurrence probabilities of exceeding the SEQS.  Figure 6 provides two priority remedy scenarios delineated from the standard-exceeding probability map based on two critical probability values 0.90 and 0.95, respectively. The suggested remedy area decreases as the selected critical probability value increases. It can be seen that most dry farmlands (except for the piece in the southeast corner) and some paddy fields (close to the south boundary) are at high risk and should be first remedied. Measures such as increasing soil pH value or transforming the farmlands into nonfarmlands may be taken to decrease the risk.

### 3.5. Discussions

As simulated realizations do not have the smoothing effect, a number of realizations may be used to explore various possible spatial patterns of the spatial variable under study (here soil Cd concentration or pH) and thus provide a visual and quantitative measure of the spatial uncertainty of the target variable. Because the standard-exceeding probability of soil Cd at a specific location was evaluated based on the land use type at the location and the simulated realizations of soil Cd concentration and pH, the spatial variability and uncertainty information of soil Cd and pH were propagated to the corresponding standard-exceeding probability. Such a standard-exceeding probability map can show where the soil is at high risk of real Cd pollution and consequently where remediation measures on Cd should be first applied.

Comparing with those studies in standard-exceeding probability estimation purely based on a single threshold value of a pollutant, the estimation in this study should be more realistic because soil pH and land use types do strongly affect the bioavailability of soil trace metals, and land use types also impact the exposure ways of trace metals to human being. Soil pH and land use type are often taken into account by environmental protection agencies in setting SEQSs which usually have the force of law. In addition, the method suggested here may also be used for estimating the standard-exceeding probability maps of other pollutants with similar restrictive conditions. However, if the considered soil properties are different or more soil properties are considered in the composite SEQS, the independence assumption among the simulated soil properties may need to be rechecked and the calculation of the joint probabilities might be complicated.

## 4. Conclusions

A method for estimating the standard-exceeding probability map of soil Cd based on a composite SEQS for Cd in soil (recently issued by China) was presented. The spatial variability and uncertainty of soil pH and site-specific land use type were incorporated through simulated realizations by SGS. The suggested method accounts for multiple Cd concentration thresholds corresponding to different soil pH value intervals and land use types. Because the composite SEQS for Cd in soil considered the effects of soil pH values and land use types, which are, respectively, related with the bioavailability (or toxicity) of Cd in soil and its human health risk through food production, the estimated standard-exceeding probability map of soil Cd using such a method can better reflect the real risk distribution of soil Cd pollution. High risk areas under two different critical probability values were further delineated for remedy consideration. The proposed method may be useful for evaluating the pollution risk of Cd in soil using a composite SEQS.

The study shows that soil Cd rarely exceeds the standard in nonfarmlands in the study area, and high risks of Cd pollution mainly occur in farmlands, particularly dry farmlands, along the interleaving zones of farmlands and nonfarmlands, where soil pH values are relatively low and industry companies are also distributed. This means that while industry may strongly impact the total concentration of Cd in soil, land use type and soil pH can be more decisive to the pollution risk of Cd in soil due to the fact that the composite SEQS considered the effects of soil pH and land use types on soil Cd bioavailability and hazard.

## Figures and Tables

**Table 1:** The soil environmental quality standard II of China for Cd in soil (mg kg^−1^).

	0 ≤ pH < 5.5	5.5 ≤ pH < 6.5	6.5 ≤ pH < 7.5	7.5 ≤ pH ≤ 14
Paddy field	0.25	0.30	0.50	1.00
Dry farmland	0.25	0.30	0.45	0.80
Nonfarmland	10.00	10.00	10.00	10.00

**Table 2:** Summary statistics of Cd concentration and pH in topsoil samples.

	Minimum	Maximum	Median	Mean	S.D.	CV (%)
Cd (mg kg^−1^)	0.26	1.94	0.68	0.74	0.32	43.22
pH	4.29	9.08	6.88	6.71	0.98	44.63

## References

[1] Lee CS-L, Li X, Shi W, Cheung SC-N, Thornton I (2006). Metal contamination in urban, suburban, and country park soils of Hong Kong: a study based on GIS and multivariate statistics. *Science of the Total Environment*.

[2] Cai L-M, Ma J, Zhou Y-Z (2008). Multivariate geostatistics and GIS-based approach to study the spatial distribution and sources of heavy metals in agricultural soil in the Pearl River Delta, China. *Huanjing Kexue*.

[3] Facchinelli A, Sacchi E, Mallen L (2001). Multivariate statistical and GIS-based approach to identify heavy metal sources in soils. *Environmental Pollution*.

[4] Zhang R, Rahman S, Vance GF, Munn LC (1995). Geostatistical analyses of trace elements in soils and plants. *Soil Science*.

[5] Goovaerts P (1997). *Geostatistics for Natural Resources Evaluation*.

[6] Juang KW, Chen YS, Lee DY (2004). Using sequential indicator simulation to assess the uncertainty of delineating heavy-metal contaminated soils. *Environmental Pollution*.

[7] Goovaerts P, Webster R, Dubois J-P (1997). Assessing the risk of soil contamination in the Swiss Jura using indicator geostatistics. *Environmental and Ecological Statistics*.

[8] Van Meirvenne M, Goovaerts P (2001). Evaluating the probability of exceeding a site-specific soil cadmium contamination threshold. *Geoderma*.

[9] Johnson M (1987). *Multivariate Statistical Simulation*.

[10] Chiles JP, Delfiner P (1999). *Geostatistics: Modeling Spatial Uncertainty*.

[11] Gaus I, Kinniburgh DG, Talbot JC, Webster R (2003). Geostatistical analysis of arsenic concentration in groundwater in Bangladesh using disjunctive kriging. *Environmental Geology*.

[12] Qu MK, Li WD, Zhang CR (2013). Assessing the spatial uncertainty in soil nitrogen mapping through stochastic simulations with categorical land use information. *Ecological Informatics*.

[13] Journel AG (1983). Nonparametric estimation of spatial distributions. *Journal of the International Association for Mathematical Geology*.

[14] Armstrong M, Matheron G (1986). Disjunctive kriging revisited: part I. *Mathematical Geology*.

[15] Gotway CA, Stroup WW (1997). A generalized linear model approach to spatial data analysis and prediction. *Journal of Agricultural, Biological, and Environmental Statistics*.

[16] De Oliveira V (2000). Bayesian prediction of clipped Gaussian random fields. *Computational Statistics and Data Analysis*.

[17] Zhao YC, Xu XH, Huang B (2007). Using robust kriging and sequential Gaussian simulation to delineate the copper- and lead-contaminated areas of a rapidly industrialized city in Yangtze River Delta, China. *Environmental Geology*.

[18] Li WD, Zhang CR, Dey DK, Wang SQ (2010). Estimating threshold-exceeding probability maps of environmental variables with Markov chain random fields. *Stochastic Environmental Research and Risk Assessment*.

[19] Mühlbachová G, Šimon T, Pechová M (2005). The availability of Cd, Pb and Zn and their relationships with soil pH and microbial biomass in soils amended by natural clinoptilolite. *Plant, Soil and Environment*.

[20] Zeng FR, Ali S, Zhang HT (2011). The influence of pH and organic matter content in paddy soil on heavy metal availability and their uptake by rice plants. *Environmental Pollution*.

[21] State Environmental Protection Administration of China (SEPAC) *Environmental Quality Standard for Soils (GB 15618-2008)*.

[22] http://www.juntadeandalucia.es/medioambiente/site/portalweb/.

[23] Qu MK, Li WD, Zhang CR (2013). Spatial distribution and uncertainty assessment of potential ecological risks of soil heavy metals using sequential gaussian simulation. *Human and Ecological Risk Assessment*.

[24] Zhao YC, Xu XH, Darilek JL, Huang B, Sun WX, Shi XZ (2009). Spatial variability assessment of soil nutrients in an intense agricultural area, a case study of Rugao County in Yangtze River Delta Region, China. *Environmental Geology*.

[25] Thompson M, Walsh JN (1983). *A Handbook of Inductively Coupled Plasma Spectroscopy*.

[26] Specialty Committee of Agricultural Chemistry (1983). *Routine Analytical Methods of Soil and Agricultural Chemistry*.

[27] Gay JR, Korre A (2006). A spatially-evaluated methodology for assessing risk to a population from contaminated land. *Environmental Pollution*.

[28] Fredericks AK, Newman KB (1998). A comparison of the sequential Gaussian and Markov-Bayes simulation methods for small samples. *Mathematical Geology*.

[29] Zhang XY, Sui YY, Zhang XD, Meng K, Herbert SJ (2007). Spatial variability of nutrient properties in black soil of northeast China. *Pedosphere*.

